# Adverse Events Related to SARS-CoV-2 Vaccine in a Nationwide Cohort of Patients With Inflammatory Bowel Disease

**DOI:** 10.14309/ctg.0000000000000554

**Published:** 2022-12-06

**Authors:** Nadim Mahmud, Walter Reinisch, Manthankumar Patel, Ramaswamy Sundararajan, Nabeel Khan

**Affiliations:** 1 Department of Gastroenterology, Corporal Michael J Crescenz VA Medical Center, Philadelphia, Pennsylvania, USA;; 2 Division of Gastroenterology, University of Pennsylvania, Perelman School of Medicine, Philadelphia, Pennsylvania, USA;; 3 Division of Gastroenterology and Hepatology, Medical University of Vienna, Vienna, Austria.

**Keywords:** SARS-CoV-2 vaccination, COVID-19, Pfizer, Moderna, inflammatory bowel disease, safety, adverse events

## Abstract

There are limited data on the safety profile of the severe acute respiratory syndrome coronavirus-2 vaccine among patients taking immunosuppressive medications. Our aim was to evaluate the adverse events related to the vaccines in a nationwide cohort of patients with inflammatory bowel disease on diverse immunosuppressive medications.

**METHODS:**

This was a retrospective cohort study using data from the Veterans Health Administration. The primary outcome was any adverse event of special interest (cerebrovascular accident, venous thromboembolism, acute myocardial infarction, Bell palsy) within 90 days of vaccination.

**RESULTS:**

A total of 17,201 patients were included, and 12,351 patients (71.8%) received at least 1 vaccine dose. The most common adverse events were acute myocardial infarction and venous thromboembolism. In inverse probability treatment weighting-adjusted logistic regression, full vaccination was not significantly associated with increased adverse events through 90 days, relative to unvaccinated patients.

**DISCUSSION:**

Full severe acute respiratory syndrome coronavirus-2 vaccination was not associated with an increased rate of key adverse events relative to unvaccinated individuals among patients with inflammatory bowel disease.

## INTRODUCTION

The severe acute respiratory syndrome coronavirus-2 (SARS-CoV-2) has affected over 596 million people worldwide as of August 24, 2022 (1). To prevent this, 2 vaccines (Pfizer BioNTech vaccine and Moderna) were initially approved by the US Food and Drug Administration.

Inflammatory bowel disease (IBD), comprising ulcerative colitis (UC), and Crohn's disease (CD), is a chronic inflammatory disorder of the gastrointestinal tract. Vaccination is strongly recommended to prevent the development of SARS-CoV-2 among patients with IBD. The vaccines have been shown to be effective among patients with IBD (2). However, despite their efficacy, a recent study in US Veteran Health Administration (VHA) cohort of patients with IBD found that only 61.8% of patients with IBD were vaccinated (3). A possible reason for this number may be the fear of adverse events reported after receipt of the SARS-CoV-2 vaccine. Our aim was to evaluate the adverse events related to the vaccines in a nationwide cohort of patients with IBD.

## METHODS

### Study design and cohort creation

This was a retrospective cohort study using data from an established national IBD cohort in the VHA. We used a previously validated algorithm based on administrative codes and pharmacy data to identify patients with UC or CD before December 18, 2020, the start date of the VHA COVID-19 vaccination campaign (index date) (3,4). Patients 18 years or older with at least 2 outpatient appointments before the index date were included. Patients were excluded if they received the Janssen vaccine during the study window given the small sample size.

### Exposures

The primary exposure in this study was SARS-CoV-2 vaccination (first dose, second dose, unvaccinated). Vaccine administration events (first and second dose, as applicable) were identified using Current Procedural Terminology codes. For each patient, we additionally obtained baseline demographics (age, sex, race), alcohol use history, tobacco use history, geographic region, IBD type (UC or CD), and Charlson Comorbidity Index. IBD medication groups in the 3 months before the index date were categorized as 5-ASA alone, thiopurines (azathioprine or mercaptopurine, i.e., thiopurines (TPs), with or without 5-aminosalicylic acid medications), antitumor necrosis factor (anti-TNF) agents alone, anti-TNF + TPs, vedolizumab, ustekinumab, and tofacitinib. Steroid use was also ascertained in a 3-month window before the index date using prescriptions for budesonide, methylprednisolone, prednisolone, or prednisone.

### Outcomes

The primary outcome was development of any adverse event of interest after SARS-CoV-2 vaccination, with follow-up data obtained through February 2, 2022. Based on observed adverse events in clinical trials, we used incident *International Classification of Diseases 9/10* codes to ascertain the first occurrence of the following composite events identified as adverse events of special interest (AESIs), which were commonly reported in the Pfizer and Moderna trials: cerebrovascular accident, venous thromboembolism (VTE), acute myocardial infarction, and Bell palsy. AESIs were identified within 90 days of first or second vaccination doses. As a reference group, AESIs for unvaccinated patients were evaluated relative to an index date set to the median time to first vaccination in patients who were vaccinated (February 14, 2021). Importantly, every adverse event was manually adjudicated for confirmation through detailed chart review. For statistical analysis, refer supplementary material.

## RESULTS

### Cohort and vaccination characteristics

After application of selection criteria and exclusion of 387 patients who received the Janssen vaccine, we identified a total of 17,201 patients with IBD. Of these, 12,351 (71.8%) received at least 1 vaccine dose and 4,850 (28.2%) remained unvaccinated (Table 1).

**Table 1.** Cohort characteristics stratified by vaccination status

Factor	Unvaccinated N = 4,850	Received vaccination (any) N = 12,351	*P* value
Age (yr), median (IQR)	59 (43, 72)	70 (58, 75)	<0.001
Age category, yr			<0.001
<65	2,872 (59.2%)	4,565 (37.0%)	
65–80	1,545 (31.9%)	6,177 (50.0%)	
>80	433 (8.9%)	1,609 (13.0%)	
Sex			0.082
Female	424 (8.7%)	980 (7.9%)	
Male	4,426 (91.3%)	11,371 (92.1%)	
Race			<0.001
White	3,869 (79.8%)	9,621 (77.9%)	
Black	532 (11.0%)	1,694 (13.7%)	
Hispanic	225 (4.6%)	587 (4.8%)	
Other	224 (4.6%)	449 (3.6%)	
Current smoker	829 (17.1%)	1814 (14.7%)	<0.001
Alcohol	2,513 (51.8%)	6,758 (54.7%)	<0.001
IBD type			0.019
Crohn's disease	1890 (39.0%)	4,576 (37.0%)	
Ulcerative colitis	2,960 (61.0%)	7,775 (63.0%)	
IBD medication group			<0.001
5-ASA only	2,646 (54.6%)	7,152 (57.9%)	
Thiopurines	533 (11.0%)	1,352 (10.9%)	
Anti-TNF	1,066 (22.0%)	2,392 (19.4%)	
Anti-TNF + thiopurines	195 (4.0%)	431 (3.5%)	
Vedolizumab	307 (6.3%)	751 (6.1%)	
Ustekinumab	58 (1.2%)	166 (1.3%)	
Tofacitinib	45 (0.9%)	107 (0.9%)	
Steroid use (baseline)	780 (16.1%)	2,298 (18.6%)	<0.001
Charlson Comorbidity Index, median (IQR)	0 (0, 1)	1 (0, 2)	<0.001
Region			<0.001
Continental	887 (18.3%)	1785 (14.5%)	
Midwest	1,201 (24.8%)	2,925 (23.7%)	
North Atlantic	1,117 (23.0%)	3,075 (24.9%)	
Pacific	692 (14.3%)	1997 (16.2%)	
Southeast	953 (19.6%)	2,569 (20.8%)	

ASA, aminosalicylic acid; IBD, inflammatory bowel disease; IQR, interquartile range; TNF, tumor necrosis factor.

### Postvaccination adverse events

A summary of postvaccination AESIs is provided in Table 2. Within 28 days of the first vaccine dose, 22 AESIs (0.18%) were recorded and 3 (0.02%) from 29 to 90 days after vaccination. After the second vaccine dose, 18 AESIs (0.15%) were noted within 28 days and 38 (0.32%) from 29 to 90 days after vaccination. From the unvaccinated index date, 4 patients (0.08%) had AESIs within 28 days and 15 (0.31%) between 29 and 90 days. The most common AESIs were VTE and acute myocardial infarction.

**Table 2.** Adverse events in postvaccination and unvaccinated groups (overall)^a^

	Days after first vaccine, n (%)		Days after second vaccine, n (%)		Unvaccinated (d), n (%)	
	0–28 (N = 12,351)	29–90 (N = 12,329)	0–28 (N = 12,043)	29–90 (N = 12,025)	0–28 (N = 4,850)	29–90 (N = 4,846)
Any adverse event	22	3	18	38	4	15
Specific adverse event						
Cerebrovascular accident	5 (0.04%)	1 (<0.01%)	6 (0.05%)	6 (0.05%)	1 (0.02%)	3 (0.06%)
Venous thromboembolism	8 (0.06%)	2 (0.02%)	7 (0.06%)	19 (0.16%)	2 (0.07%)	9 (0.19%)
Acute myocardial infarction	8 (0.06%)	0 (0.00%)	4 (0.03%)	12 (0.10%)	1 (0.02%)	3 (0.06%)
Bell palsy	1 (<0.01%)	0 (0.00%)	1 (<0.01%)	0 (0.00%)	0 (0.00%)	0 (0.00%)

Logistic regression models for any 90-d adverse events				
	First vaccine dose vs unvaccinated		Second vaccine dose vs unvaccinated	
	OR (95% CI)	*P* value	OR (95% CI)	*P* value
Unadjusted	0.52 (0.28–0.94)	0.03	1.25 (0.74–2.11)	0.39
IPTW-adjusted	0.38 (0.26–0.57)	<0.001	0.86 (0.63–1.18)	0.36

CI, confidence interval; IPTW, inverse probability treatment weighting; OR, odds ratio.

a Note that total sample sizes only include the number at risk of developing an adverse event (i.e., excluding those who had already developed an adverse event of interest). Most patients contributed follow-up time to both unvaccinated and vaccinated groups.

### Association between vaccination and adverse events

After creation of propensity scores and application of inverse probability weights, excellent covariate balance was achieved between unvaccinated and vaccinated groups, demonstrated by standardized mean differences reduced to within ± 0.1 for each exposure variable (see Supplemental Figure 1, Supplementary Digital Content, http://links.lww.com/CTG/A895). In unadjusted models, first-dose vaccination was associated with reduced odds of AESIs vs unvaccinated patients (odds ratio [OR] 0.52, 95% confidence interval [CI] 0.28–0.94, *P* = 0.03), and there was no significant difference in AESIs between second-dose vaccination and unvaccinated patients (OR 1.25, 95% CI 0.74–2.11, *P* = 0.39; Table 2). Similar results were observed in inverse probability treatment weighting-adjusted models: OR 0.38, 95% CI 0.26–0.57, *P* < 0.001 for first dose versus unvaccinated and OR 0.86, 95% CI 0.63–1.18, *P* = 0.36 for second dose versus unvaccinated. In a secondary analysis where recent SARS-CoV-2 infection was added to the model for second-dose vaccination, the effect estimates for vaccination remained essentially unchanged (OR 0.86, 95% CI 0.63–1.18, *P* = 0.34).

## DISCUSSION

In the Pfizer vaccine trial, the serious adverse events which were higher among those who received the vaccine vs placebo were acute myocardial infarction and cerebrovascular accident (5). For the Moderna vaccine, these were Bell palsy, myocardial infarction, and nephrolithiasis (6). Because patients with IBD have an inherently increased risk of developing myocardial infarction (7) as well as VTE (8), it was reassuring that fully vaccinated state was not associated with an increased risk of these events.

Major strengths of this work include the use of a large, national cohort of patients with IBD taking diverse medication regimens and an inverse probability weighted design to account for variation in baseline characteristics between vaccinated and unvaccinated individuals. Each adverse event was confirmed by individual review of the chart. The primary limitation is that the cohort comprised an older predominantly male population, which may hinder broad generalizability of the results.

In conclusion, our study showed that among patients with IBD, full vaccination state is not associated with an increased risk of adverse events attributable to vaccination. Furthermore, the overall rate of adverse events was very low. This is of relevance given the large proportion of patients who remain unvaccinated and hopefully will lead to increased vaccine adoption.

## CONFLICTS OF INTEREST

**Guarantor of the article:** Nabeel Khan, MD.

**Specific author contributions:** N.K. has participated in study supervision, study concept and design, acquisition of data, analysis and interpretation of data, drafting of the manuscript, and critical revision of the manuscript for important intellectual content. N.M. has participated in study concept and design, acquisition of data, formal statistical analysis, data visualization, analysis and interpretation of data, and drafting of the manuscript. W.R. has participated in study concept and design, analysis and interpretation of data, drafting of the manuscript, and critical revision of the manuscript for important intellectual content. M.P. has participated in study concept and design, acquisition of data, interpretation of data, and drafting of the manuscript. R.S. has participated in study concept and design, acquisition of data, interpretation of data, and drafting of the manuscript.

**Financial support:** N.M. is supported by an American College of Gastroenterology Junior Faculty Development Award (ACG-JR-010-2020) and by the National Institute of Diabetes and Digestive and Kidney Diseases (K08-DK124577). There was no designated funding received for this study.

**Potential competing interests:** N.K. has received an unrestricted research grant from Pfizer, Luitpold, and Takeda Pharmaceuticals as well as Samsung BioEpis. He has served on the advisory board of Pharmacosmos. N.M., M.P., and R.S. have nothing to disclose regarding conflicts of interest. W.R. (i) has served as a speaker for Abbott Laboratories, AbbVie, Aesca, Aptalis, Astellas, Centocor, Celltrion, Danone Austria, Elan, Falk Pharma GmbH, Ferring, Immundiagnostik, Mitsubishi Tanabe Pharma Corporation, MSD, Otsuka, PDL, Pharmacosmos, PLS Education, Schering-Plough, Shire, Takeda, Therakos, Vifor, and Yakult; (ii) has served as a consultant for Abbott Laboratories, AbbVie, Aesca, Algernon, Amgen, AM Pharma, AMT, AOP Orphan, Arena Pharmaceuticals, Astellas, AstraZeneca, Avaxia, Roland Berger GmBH, Bioclinica, Biogen IDEC, Boehringer-Ingelheim, Bristol-Myers Squibb, Cellerix, Chemocentryx, Celgene, Centocor, Celltrion, Covance, Danone Austria, *DSM*, Elan, Eli Lilly, Ernst & Young, Falk Pharma GmbH, Ferring, Galapagos, Genentech, Gilead, Grünenthal, ICON, Index Pharma, Inova, Intrinsic Imaging, Janssen, Johnson & Johnson, Kyowa Hakko Kirin Pharma, Lipid Therapeutics, LivaNova, Mallinckrodt, Medahead, MedImmune, Millennium, Mitsubishi Tanabe Pharma Corporation, MSD, Nash Pharmaceuticals, Nestle, Nippon Kayaku, Novartis, Ocera, OMass, Otsuka, Parexel, PDL, Periconsulting, Pharmacosmos, Philip Morris Institute, Pfizer, Procter & Gamble, Prometheus, Protagonist, Provention, Quell Tx, Robarts Clinical Trial, Sandoz, Schering-Plough, Second Genome, Seres Therapeutics, Setpointmedical, Sigmoid, Sublimity, Takeda, Therakos, Theravance, Tigenix, UCB, Vifor, Zealand, Zyngenia, and 4SC; (iii) has served as an advisory board member for Abbott Laboratories, AbbVie, Aesca, Amgen, AM Pharma, Astellas, Astra Zeneca, Avaxia, Biogen IDEC, Boehringer-Ingelheim, Bristol-Myers Squibb, Cellerix, Chemocentryx, Celgene, Centocor, Celltrion, Danone Austria, *DSM*, Elan, Ferring, Galapagos, Genentech, Grünenthal, Inova, Janssen, Johnson & Johnson, Kyowa Hakko Kirin Pharma, Lipid Therapeutics, MedImmune, Millennium, Mitsubishi Tanabe Pharma Corporation, MSD, Nestle, Novartis, Ocera, Otsuka, PDL, Pharmacosmos, Pfizer, Procter & Gamble, Prometheus, Sandoz, Schering-Plough, Second Genome, Setpointmedical, Takeda, Therakos, Tigenix, UCB, Zealand, Zyngenia, and 4SC; and (iv) has received research funding from Abbott Laboratories, AbbVie, Aesca, Centocor, Falk Pharma GmbH, Immundiagnostik, and MSD.

**Data Availability Statement:** The data for this manuscript cannot be made available in accordance with the HIPAA rules. However, deidentified data (without patient name and SSN) can be made available upon reasonable request.

**IRB Approval Statement:** Data were collected using preexisting electronic patient records in the VISTA CAPRI database after an institutional review board (IRB) approval from the Corporal Michael J Crescenz VA Medical Center, Philadelphia, PA.

## Supplementary Material

SUPPLEMENTARY MATERIAL
