# Supplementary material for: Adverse Events Related to SARS-CoV-2 Vaccine in a Nationwide Cohort of Patients With Inflammatory Bowel Disease
Source: Clin Transl Gastroenterol. 2022 Dec 6;14(4):e00554. doi: 10.14309/ctg.0000000000000554 (PMC10132715; doi:10.14309/ctg.0000000000000554)

**Supplemental material:**

*Statistical Analysis*

Descriptive statistics for the cohort were summarized using medians and interquartile ranges (IQRs) for continuous data and percentages for categorical data. Exposure variables were stratified by vaccination status (at least one dose received versus unvaccinated), and comparisons made using Wilcoxon rank-sum and chi-squared tests, as indicated. The raw proportions of adverse events occurring within 0-28 days and 29-90 days of first dose, second dose, or the unvaccinated index date were presented. Proportions were only computed for the number of individuals at risk of AESI (i.e., excluding those who had already developed an AE of interest).

To account for underlying differences in patients who received vaccination and those who were unvaccinated, we used an inverse probability treatment weighting (IPTW) approach. We generated a propensity score (PS) for receiving vaccination (any) using all available exposure variables as predictors. We then computed inverse probability weights as 1/PS for vaccinated individuals and 1/(1-PS) for unvaccinated individuals **[1]**. We plotted the standardized mean differences (SMD) for unadjusted and IPW-adjusted variables, where an SMD between +/- 0.1 was regarded to reflect excellent covariate balance. This approach is conceptually similar to recently published work from our group using this IBD cohort **[2].**

In order to evaluate the risk of AESI attributable to vaccination, we used a logistic regression approach. We created unadjusted and IPTW-adjusted models to evaluate the odds ratio (OR) and 95% confidence interval (CI) of AESI for first and second vaccination doses, each relative to an unvaccinated status. In a secondary analysis, we identified SARS CoV-2 infections in the 90 days prior to index date, and added this covariate to the IPTW-adjusted models. For all hypothesis tests, an alpha threshold of 5% was used to determined statistical significance.

References:

1. Austin PC, Stuart EA. Moving towards best practice when using inverse probability of treatment weighting (IPTW) using the propensity score to estimate causal treatment effects in observational studies. Statistics in medicine 2015;34:3661-3679.
2. Khan N, Mahmud N. Effectiveness of SARS-CoV-2 Vaccination in a Veterans Affairs Cohort of Patients With Inflammatory Bowel Disease With Diverse Exposure to Immunosuppressive Medications. Gastroenterology. 2021 Sep;161(3):827-836. doi: 10.1053/j.gastro.2021.05.044. Epub 2021 May 25. PMID: 34048782; PMCID: PMC8146263.

Supplemental Figure 1: Unadjusted and Inverse Probability Weights-Adjusted variables


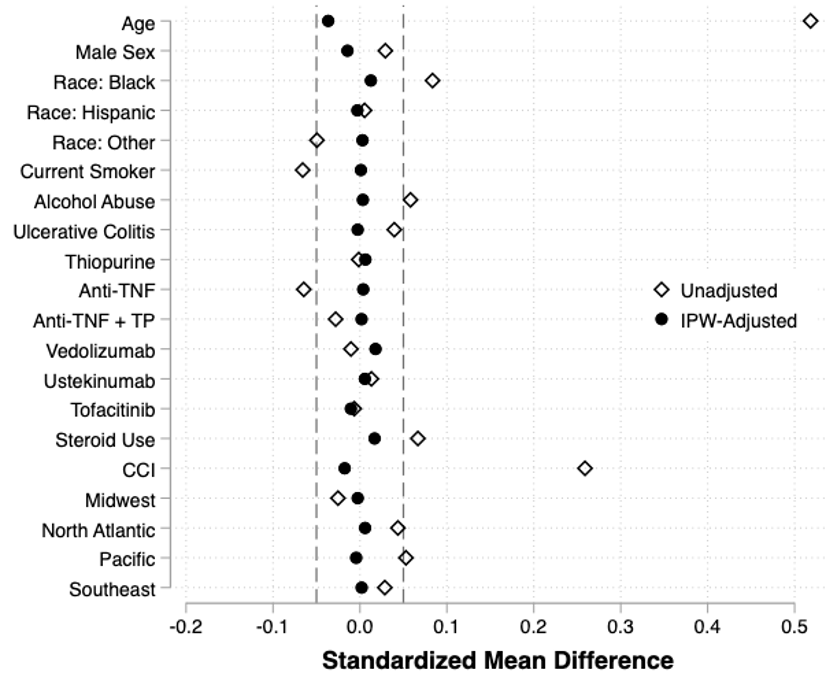

Supplement: SUPPLEMENTARY MATERIAL [file ct9-14-e00554-s001.docx]
